# Virtual visits to inpatients by their loved ones during COVID-19

**DOI:** 10.6061/clinics/2020/e2171

**Published:** 2020-07-15

**Authors:** Izabel Cristina Rios, Ricardo Tavares de Carvalho, Vitor Maia Teles Ruffini, Amanda Cardoso Montal, Leila Suemi Harima, Douglas Henrique Crispim, Lilian Arai, Beatriz Perondi, Anna Miethke Morais, Andrea Janaina de Andrade, Eloisa Silva Dutra de Oliveira Bonfa

**Affiliations:** Hospital das Clinicas HCFMUSP, Faculdade de Medicina, Universidade de Sao Paulo, Sao Paulo, SP, BR

As a consequence of the SARS-CoV-2 pandemic, social contact among people has undergone significant changes. To slow the spread of the virus, social distancing measures, such as isolation and the maintenance of distance between individuals, which includes changing the manner in which people greet each other, are in place, demonstrating responsible health practices. The lack of physical contact with other human beings is a major challenge during this highly contagious pandemic.

To protect vulnerable people, hospitals have discontinued all visitations; so, from the moment a patient is admitted to the hospital, he or she is alone. COVID-19 is a painful disease because in addition to the intense physical suffering, there is an added, unprecedented, emotional strain because patients cannot receive support from their families. This separation leads to feelings of solitary agony and helplessness, not only for patients but also for their family members (1).

Across the world, healthcare providers have tried to diminish the loneliness of patients and families by enabling the use of remote communication devices (2). As many patients are elderly, or incapacitated, they are unable to use the equipment themselves, and so nurses and doctors have developed the idea of virtual visits (3). Virtual visits are meetings between patients and their family members using technological communication means.

The importance of this process was emphasized by the claims of healthcare providers that although a medical visit (4) was essential to keep families informed about the clinical status of their relatives who were admitted to the hospital requests were often made to see and talk to the patients and that even a very short visit brought significant comfort to the patients. Moved by compassion, healthcare providers initially used their own devices to bring patients closer to their loved ones; subsequently, this became an institutional practice of care. It was observed that patients and families shared more than just information during these visits—they received encouragement from and supported each other to help cope with the suffering and disease (5,6).

Despite the benefits of the virtual visit for patients and their relatives, its implementation is not easy as healthcare staff is usually overburdened during a pandemic and thus is under stress and is more focused on medical treatment than on care. To overcome this problem, in the Hospital das Clínicas, School of Medicine of the University of São Paulo, we initiated a model to implement virtual visits with a team of volunteers not directly involved in patient care. This strategy allowed us to enable virtual visits to our patients in the wards as part of integral assistance during the hospitalization period without overburdening the healthcare staff dedicated to providing medical treatment and assistance. Such virtual visits have demonstrated very good outcomes for patients, families, and volunteers.

The first obstacle for implementing virtual visits was the lack of a sufficient number of devices for the 46 wards, including Emergency Rooms and Intensive Care Units. We chose to use tablets because of the following advantages: allows the viewing of larger and more accurate images, allows the participation of more than one family member, and allows making videocalls to family members via WhatsApp, the most widely used communication resource in Brazil. All devices were donated by one communications technology company.

Another major concern was the risk of the disease spreading to the volunteers. To overcome this problem, the blood pressure monitor floor stand model was altered to create a metal stand support with wheels, and the tablet was attached onto this modified device. This solution enabled a reduction in the exposure of the volunteers to the virus as they can place this modified stand with the tablet in front of the patient and then move away from the bed. Figure 1 shows the tablet support device.

The challenge involved in engaging a team of volunteers was solved by online dissemination of the project to medical students, who had the right mindset for this task. Twenty-eight current and former medical students joined the virtual visit team (VVT). They were trained in family and patient communication skills, and they also received orientation regarding COVID-19 safety protocols.

The Humanization Group (HG) of the Hospital da Clínicas led the virtual visits project. Although virtual visits are a relatively simple idea, the operational logistics involved in connecting patients with families requires attention to various details. Herein, we present the step-by-step routine to be followed:

1. The healthcare team selects patients who will participate in the virtual visit by videocall or by audio or video recording, prioritizing those who are too incapacitated to use their own device. Videocalls are more suitable for patients to be able to speak and interact with their families. Audio or video recordings are preferred when patients are unconscious or cannot communicate. The healthcare team asks patients for their consent to be involved in the virtual visit.
2. The healthcare team adds the patients’ data in a spreadsheet, which is shared with the HG.
3. Members of the HG team contact the patients’ families by telephone to explain to them how the virtual visits will work and also list the rules to be followed: they do not have permission to photograph or record the visit; patient health information will be not provided during this procedure (this was because the healthcare team would schedule a daily call, providing updates regarding the clinical status of the patients), and the volunteer will stay near the patient during the conversation. If these conditions were agreed to, further steps were considered.
4. The HG team receives the volunteer schedule for the day and gives the volunteers instructions about the patients scheduled for virtual visits. Then, the volunteers head for the wards. Each volunteer carries out his/her task in 1 or 2 wards, visiting about 6 to 8 patients.
5. In the ward, the volunteer collects the tablet and finishes the safety procedures required for the area.
6. In the patient’s rooms, the volunteer confirms the patient’s willingness to participate in the virtual visit, then connects the call via WhatsApp. The rules of the virtual visit are explained once more, and then the volunteer allows the patient to speak for 10 minutes with his/her family. After this time has passed, the volunteer asks if there are any doubts, says goodbye and ends the call. Then, the volunteer blocks the cell phone number called for avoiding the inadequate use by family.
7. At the end of day, the volunteers clean the equipment and return it to the wards.
8. The volunteers register the visit in an electronic medical record and a control data spreadsheet. Figures 2 and 3 illustrate the virtual visit.

Over the course of one month, the VVT undertook 234 virtual visits. The patients, families, and volunteers perceived the virtual visits as beneficial. The patients and their families were reported to be appreciative of the chance to see and communicate with each other. The virtual visit allows for the meeting between loved ones, which enhances the health of the patient, provides emotional comfort and a form of contact, and increases the hope of healing. As virtual visits promote emotional well-being, they will soon become part of a set of measures geared toward improving the quality of healthcare during the COVID-19 pandemic. The volunteers also reported a positive impact and felt personal satisfaction from their contribution. In addition, the virtual visit constitutes an innovation that can be adopted by healthcare organizations for any other situation in which patients cannot physically meet their loved ones.

Finally, the following list provides some tips to help develop virtual visits in a healthcare service:

- For safety reasons, only hospital devices are recommended for use.
- Wireless connection may be a problem, and assuring its good performance is essential.
- Time should be spent in selecting volunteers who really want to engage in this process and have some familiarity with the hospital environment.
- Logistical support is needed by the VVT, in addition to leaders who can monitor the process and be reached at all times.
- Exceptionally patients and families have conflicts between them during the virtual visit. In this case, volunteers must be prepared to facilitate the end of the visit.
- Debriefing sessions for exchanging experiences among VVT members are essential for empowerment and for the volunteers to remain motivated to continue participating in the project.

Notwithstanding the hard work that the development of virtual visits requires, we provide here a concrete example of the use of technology in parallel with compassion that together make patient care more compassionate and effective.

## Figures and Tables

**Figure 1:**
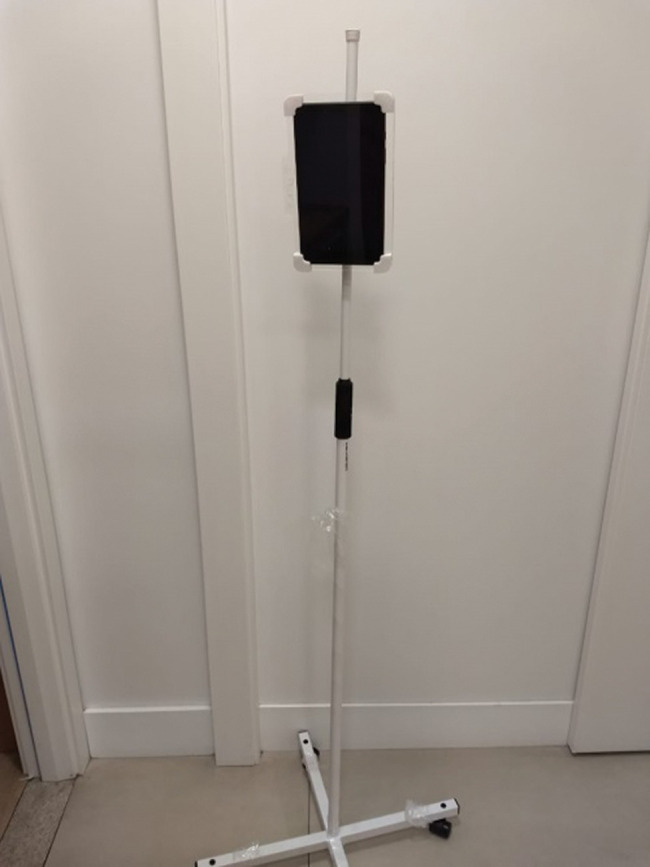
Tablet support device. Photo by Andrea J. Andrade

**Figure 2:**
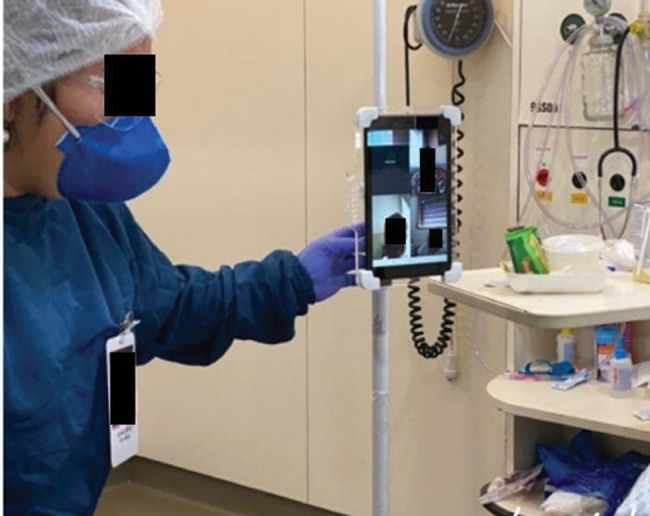
Virtual visit. Photo by Stela Murgel

**Figure 3:**
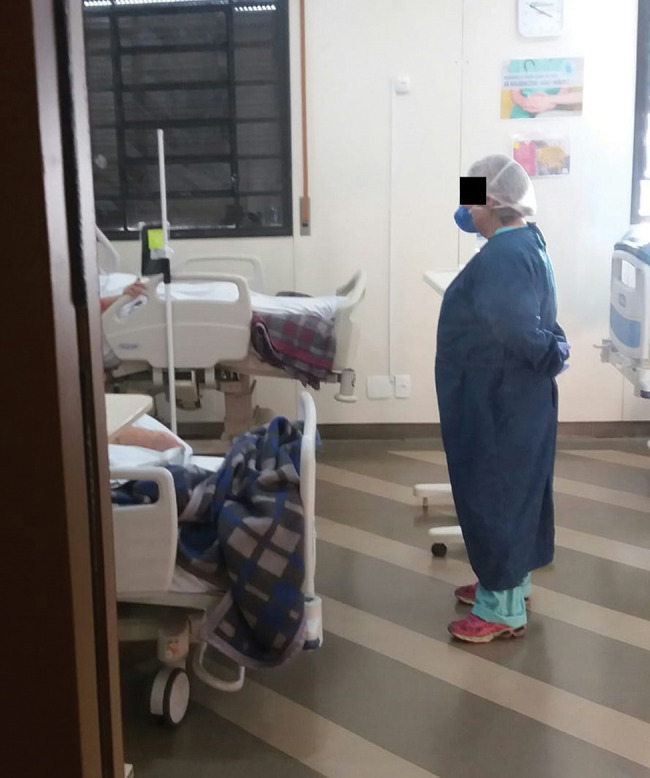
Virtual visit. Photo by Andrea J. Andrade
